# Selective treatment pressure in colon cancer drives the molecular profile of resistant circulating tumor cell clones

**DOI:** 10.1186/s12943-021-01326-6

**Published:** 2021-02-08

**Authors:** Laure Cayrefourcq, Frédéric Thomas, Thibault Mazard, Eric Assenat, Said Assou, Catherine Alix-Panabières

**Affiliations:** 1 Laboratory of Rare Human Circulating Cells, University Medical Center of Montpellier, University of Montpellier, Montpellier, France; 2 CREEC (CREES), Unité Mixte de Recherches, IRD 224–CNRS 5290–University of Montpellier, Montpellier, France; 3 Department IRCM, Inserm, University of Montpellier, ICM, Montpellier, France; 4 Department of Medical Oncology, University Medical Center of Montpellier, University of Montpellier, Montpellier, France; 5 IRMB, University of Montpellier, INSERM, CHU Montpellier, Montpellier, France

**Keywords:** Circulating tumor cells, Gene expression, Clonal evolution, Colon cancer, CDA, ALDOB

## Abstract

The characterization of circulating tumor cells (CTCs) holds promises for precision medicine because these cells are an important clinical indicator of treatment efficacy. We established the first and still only nine permanent colon CTC lines from peripheral blood samples of a patient with metastatic colon cancer collected at different time points during treatment and cancer progression. The study objectives were (*i*) to compare the gene expression profiles of these CTC lines, and (*ii*) to determine the main features acquired during treatment. The number of upregulated genes was higher in the CTC lines obtained after treatment, indicating that they acquired properties to escape treatment pressure. Among these upregulated genes, some are involved in the mTOR and PI3K/AKT signaling pathways. Moreover, cytidine deaminase expression was significantly increased in the CTC lines obtained after failure of the first- and second-line 5-fluorouracile-based treatments, suggesting that these CTCs can eliminate this specific drug and resist to therapy. Several enzymes involved in xenobiotic metabolism also were upregulated after treatment, suggesting the activation of detoxification mechanisms in response to chemotherapy. Finally, the significant higher expression of aldolase B in four of the six CTC lines obtained after treatment withdrawal and cancer progression indicated that these clones originated from liver metastases. In conclusion, these CTC lines generated at different time points during treatment of metastatic colon cancer in a single patient are characterized by the deregulation of different genes that promote (*i*) drug resistance, (*ii*) xenobiotic and energy metabolism, and (*iii*) stem cell properties and plasticity.

**Supplementary Information:**

The online version contains supplementary material available at 10.1186/s12943-021-01326-6.

## Main text

Liquid biopsy-based strategies may transform oncology in the near future [1]. Many studies analyzed circulating tumor cells (CTCs) as a real-time liquid biopsy in epithelial tumors (i.e. breast, prostate, and colon cancer) [2] and showed that their study contributes to the prognostic evaluation, patient stratification, and real-time monitoring of treatment efficacy, as well as to the identification of therapeutic targets and tumor resistance mechanisms. However, the low CTC concentration in blood is a crucial limiting factor for the identification of CTCs that may initiate a new tumor at a distant site (i.e. “metastasis-initiator CTCs” or “metastasis-competent CTCs”). Moreover, the establishment and long-term maintenance of in vitro CTC cultures are a major challenge, achieved only by few groups [3, 4].

We established the first nine permanent CTC lines from a 57-year-old patient with metastatic colon cancer (MCC). At diagnosis, he had unresectable widespread MCC with abdominal and mediastinal lymph node invasion and liver metastases. The patient was first treated with the 5-fluorouracile (5-FU)-irinotecan (FOLFIRI) combination and bevacizumab (5 cycles). The first biological progression was observed after the fifth FOLFIRI cycle, and second-line treatment was initiated with 5-FU-oxaliplatin (FOLXFOX) and bevacizumab, but was stopped after the third cycle because of disease progression (clinical and morphological). The patient died about 6 months after diagnosis due to cancer progression. We derived these CTC lines from blood samples collected before treatment initiation (CTC-MCC-41) [5], after the first- and second-line treatments (CTC-MCC-41.4), and 1 week before the patient’s death (CTC-MCC-41.5 [A-G]) [6]. Their phenotypic and molecular characterization indicated that they present common traits and all display epithelial-to-mesenchymal plasticity and stem cell-like characteristics [6, 7].

Here, we obtained and compared the transcriptomic profiles of these CTC lines to identify expression changes linked to tumor progression and treatment pressure, with the aim of identifying biomarkers and mechanisms involved in colon cancer progression.

## Results and discussion

### CTC selection during cancer progression and treatment pressure

**Fig. 1:**
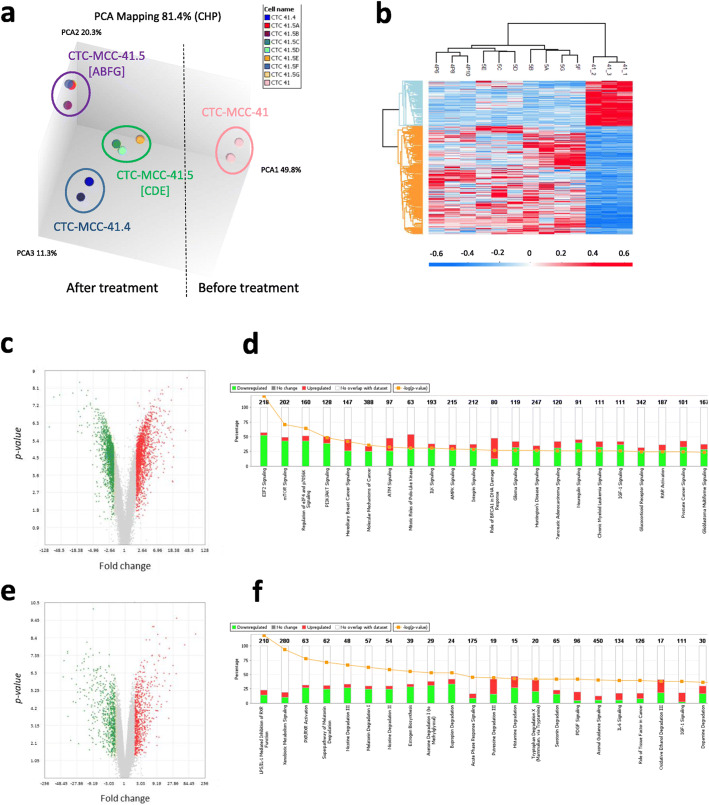
Transcriptomic analysis of the nine CTC lines obtained from a patient with metastatic colon cancer at different times during disease progression. **a** Unsupervised 3D PCA representing the gene expression patterns of the CTC line obtained before treatment (*n* = 3 samples) and the eight CTC lines obtained after treatment initiation (*n* = 10 samples); **b** Hierarchical clustering of the CTC lines based on the differentially expressed transcripts (DETs) in CTC-MCC-41 (before treatment) and the other eight lines. The color intensity indicates the transcript expression level (red for upregulated transcripts and blue for downregulated transcripts); **c** Volcano plots showing the distribution of gene expression fold changes and *p-*values using the TAC software. Transcripts upregulated in CTC-MCC-41 are indicated in green, and transcripts upregulated in the other eight CTC lines are in red. **d** Top enriched pathways for the DETs (ANOVA analysis), ranked in increasing order (Fisher’s exact test *p-*value) identified by Ingenuity® Pathway Analysis, by comparing CTC-MCC-41 (before treatment) and the other eight lines. The “EIF2 signaling”, “mTOR and “PI3K/AKT signaling” pathways were significantly deregulated. Red: genes upregulated in CTC-MCC-41 compared with the other eight CTC lines; green: genes upregulated in the eight post-treatment CTC lines; white: no overlap with the data set; Orange - log (*p*-value); **e** Volcano plots showing the distribution of gene expression fold changes and *p-*values using the TAC software, with transcripts upregulated in the CTC-MCC-41.5 [ABFG] subgroup (green) and in the CTC-MCC-41.5 [CDE] subgroup (red). **f** Top enriched pathways for the DETs based on the ANOVA analysis, ranked in increasing order (Fisher’s exact test *p-*values) identified by Ingenuity® Pathway Analysis, in the comparison of the seven CTC-MCC-41.5 lines obtained just before the patient’s death. Many “metabolism” pathways were significantly deregulated, including “xenobiotic metabolism signaling”. Abbreviations: TAC, Transcriptome Analysis Console; DET, differentially expressed transcripts

The analysis of the transcriptomic profiles of the nine CTC lines using Affymetrix HG-U133P microarray chips clearly separated them in four distinct groups: CTC-MCC-41, CTC-MCC-41.4, CTC-MCC-41.5 [ABFG], and CTC-MCC-41.5 [CDE] (Fig. 1a). We then compared the transcriptomic profiles of the pre-treatment CTC-MCC-41 line and of the other eight CTC lines (CTC-MCC-41.4 and CTC-MCC-41.5[A-G]) to understand the treatment impact on CTC clonal selection (Fig. 1b and Additional file 2*:* Tables S1–2). The significantly higher number of upregulated genes in the eight cell lines obtained after treatment initiation (Fig. 1c) suggests that they acquired properties to adapt and resist to treatment. For instance, genes involved in the mTOR and PI3K/AKT signaling cascades, which are implicated in cancer development by coordinating cell growth, survival and proliferation, and in resistance to chemotherapy, were upregulated in the post-treatment CTC lines (Fig. 1d). Colon cancer-specific mortality is higher in patients with tumors harboring mutated PIK3CA than wild type PIK3CA [8]. All our CTC lines harbored wild type PIK3CA and AKT [6], but these signaling pathways were deregulated. Some studies demonstrated that PI3K/mTOR pathway inhibitors could be used in primary and metastatic colorectal cancer [9]. We recently reported that CTC-MCC-41 cells also respond to mTOR and AKT inhibitors, suggesting these therapies are effective even in the absence of mutations [10].

Conversely, we did not observe many differences between the CTC-MCC-41.4 line, obtained after the last treatment, and the seven CTC-MCC-41.5 [A-G] lines obtained before the patient’s death (Additional file 2: Table S3). This suggests that, without drug pressure, the rapid cancer worsening was not link anymore to a clonal evolution but is due to natural disease progression with the replication of the CTC clones already selected under treatment. Indeed, the seven last CTC lines seem to be already present in the pooled CTC-MCC-41.4 line and they have been selected in the in vitro culture from the last blood sample. However, we could clearly segregate the last seven CTC cell lines (CTC-MCC-41.5 [A-G]), in two groups, [ABFG] and [CDE] (Fig. 1a), with different gene expression profiles (Fig. 1e). Most of the significantly deregulated pathways were involved in metabolism signaling (Fig. 1f), including xenobiotic metabolism. This suggests that detoxification mechanisms were induced upon exposure to anti-cancer drugs, as indicated by the deregulation of the irinotecan/SN38 pathway specifically in the [CDE] group. Furthermore, lipid metabolism upregulation appeared to be more represented in the “CDE signature”. Lipid metabolism is a key function on the basis of the enrichment of different signaling cascades leading to energy metabolism. Since Warburg’s work, metabolic reprogramming is one of the main hallmarks of cancer cells and plays a critical role in the continued tumor growth and progression and is driven by a complex interplay between the tumor mutational landscape, epigenetic modifications, and microenvironmental influences [11]. This topic is actively studied and a high-throughput metabolic-based assay was developed for rapid detection of rare metabolically active disseminated tumor cells in pleural effusion of lung cancer [12].

### Cytidine deaminase as a drug resistance biomarker

**Fig. 2:**
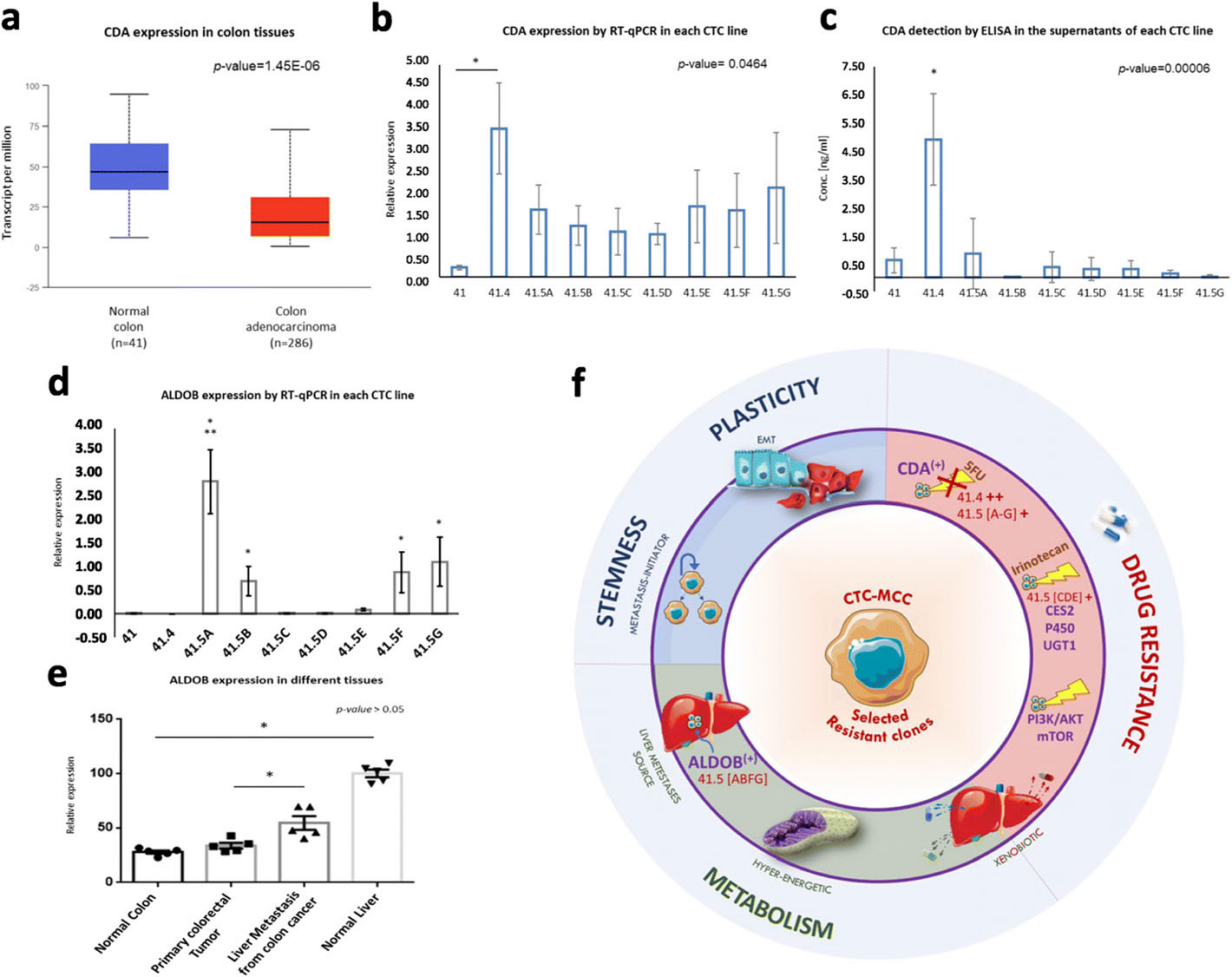
Major features of the aggressive colon cancer CTC clones selected after treatment. **a** Boxplot showing cytidine deaminase (*CDA*) relative expression in normal colon (*n* = 41) and colon adenocarcinoma (*n* = 286) samples using UALCAN (*p-value = 1.45E-06*). **b** RT-qPCR analysis of *CDA* gene expression in the nine CTC lines (mean values of triplicate experiments with standard deviation). **c** ELISA measurement of CDA protein level (ng/ml) in the conditioned medium from the nine CTC lines (mean values from triplicate experiments with standard deviation). Aldolase B (*ALDOB*) expression analysis (**d**) by RT-qPCR in the nine CTC lines [*significant upregulation in the CTC-MCC-41.5 [ABFG] cell lines compared with all the other CTC lines (*p-value = 0.0134),* and only with the CTC-MCC-41.5 [CDE] lines (*p-value = 0.0466*); **significantly upregulation compared with all the other CTC lines (*p-value = 0.0017*)], and **e** in tissue samples (normal colon, normal liver, primary colorectal tumor, liver metastases of colon cancer) (*n* = 5/tissue). **f** Representation of the cancer hallmarks of the CTC lines resistant to treatment: (*i*) Stemness and Plasticity: metastasis-competent potential, (*ii*) Drug resistance: different molecules and pathways involved in treatment resistance and drug detoxification, such as the CDA enzyme implicated in pyrimidine analogue metabolism (e.g. for 5-FU), (*iii*) Metabolism: *xenobiotic metabolism* linked to therapy resistance, *energy metabolism* linked to mitochondrial activity, and *ALDOB activity* that gave information also on the liver metastasis origin of the CTC41.5[ABFG] lines. Abbreviation: EMT, epithelial-mesenchymal transition

Besides deregulation of the irinotecan/SN38 pathway in the [CDE] group, the gene encoding cytidine deaminase (CDA) was deregulated in the post-treatment CTC lines (Additional file 2: Table S2). This finding in a patient treated with 5-FU, a pyrimidine analogue, is interesting because CDA maintains the cellular pyrimidine pool by catalyzing the hydrolytic deamination of cytidine and deoxycytidine to uridine and deoxyuridine. Some studies showed that in patients treated with direct cytidine analogues, such as gemcitabine and cytosine arabinoside, CDA overexpression might be a marker of resistance [13]. Analysis of *CDA* expression in colorectal cancer using publicly available data [14] indicated that it was significantly downregulated in colon adenocarcinoma compared with normal colon (Fig. 2a). However, *CDA* was strongly upregulated in the CTC-MCC41.4 cell line obtained directly after failure of the first- and second-line 5-FU-based treatments (RT-qPCR analysis in Fig. 2b) and also, to a lower extent, in the CTC lines (CTC-MCC-41.5 A-G) obtained just before death, compared with the pre-treatment CTC-MCC-41 line.

As CDA is secreted in the extracellular compartment (https://www.uniprot.org/uniprot/P32320) and can be detected in blood, we quantified CDA concentration by ELISA in conditioned medium from the nine CTC-MCC lines. As observed for the *CDA* gene, CDA protein level was highest in CTC-MCC-41.4 cells (Fig. 2c). These findings suggest that in this patient, CDA was directly produced and secreted by resistant and aggressive CTCs in response to 5-FU-based chemotherapy. Therefore, CDA might represent a candidate plasmatic biomarker to monitor 5-FU efficacy and resistance development.

### Aldolase B as a marker to identify CTCs released only by liver metastases

Comparison of the signatures of the two CTC-MCC-41.5 sub-groups ([ABFG] and [CDE]) (Additional file 2*: Tables S4–5*) highlighted *ALDOB* upregulation in the [ABFG] group (Fig. 2d). This gene encodes the aldolase B enzyme (fructose-bisphosphate aldolase B or liver-type aldolase), one of three isoenzymes (A, B, and C) of the class I fructose 1,6-bisphosphate aldolase enzyme (EC 4.1.2.1) that plays a key role in glycolysis and gluconeogenesis. This enzyme is preferentially expressed in the liver and at lower extend in the kidney and the small intestine. *ALDOB* downregulation correlates with poor overall survival in liver and gastric cancer [15, 16], whereas *ALDOB* overexpression in colorectal cancer has been associated with poor overall survival and epithelial-to-mesenchymal transition promotion [17]. Colorectal cancer is known to preferentially metastasize (∼70% patients) to the liver [18], which is the main organ for glycogenesis and gluconeogenesis. In addition, this specific patient had liver metastases. Comparison of *ALDOB* expression in different tissues using publicly available data showed that: (*i*) *ALDOB* was strongly expressed in normal liver compared with normal colon, (*ii*) *ALDOB* expression was comparable in primary colon adenocarcinoma and normal colon samples, and (*iii*) *ALDOB* was upregulated in colon cancer liver metastases compared with primary tumors (Fig. 2e). Bu et al. showed that during liver colonization, colon cancer cells undergo metabolic reprogramming by upregulating *ALDOB* [19]. This enhances fructose metabolism and promotes the growth of colon cancer liver metastases. These data suggest that the [ABFG] lines were derived from CTCs released by liver metastases. It is thought that CTCs are released by the primary tumor and/or metastases; however, to our knowledge, this is the first time that this could be demonstrated in a patient.

## Conclusions

From Nowell’s work in 1976, it is acknowledged that cancer is an evolutionary process and that treatments exert selective pressures that drive the tumor cell evolution, favoring the appearance of resistant clones. The present results confirm that CTC line profiling is a relevant approach to study clonal selection during disease progression and to discover new CTC biomarkers for monitoring treatment response. The transcriptomic analysis of the CTC lines obtained after treatments (CTC-MCC-41.4 and CTC-MCC-41.5[A-G]) showed the progressive deregulation of genes involved in cancer aggressiveness hallmarks: (*i*) drug resistance, with the upregulation of molecules and pathways implicated in drug detoxification (e.g. CDA and the irinotecan/SN38 pathway), (*ii*) metabolism changes, including upregulation of genes implicated in the xenobiotic metabolism, linked to therapy resistance, and energy metabolism (mitochondrial activity to provide energy to hyper-metabolic cells), and the ALDOB gene linked to liver metabolism, and (*iii*) stemness and plasticity, highlighting their metastasis-competent potential (Fig. 2f). This work is, however, specific of this cancer patient and the biomarkers found and highlighted in these CTC lines need now to be validated on CTCs and plasma from independent patients with colorectal cancer.

## Supplementary Information

**Additional file 1.** Supplementary materials and methods.

**Additional file 2: Table S1.** List of DETs upregulated in CTC-MCC-41 vs all the other CTC lines. **Table S2.** List of DETs downregulated in CTC-MCC-41 vs all the other CTC lines. **Table S3.** List of DETs upregulated in all CTC-MCC-41.5 cell lines vs the CTC-MCC-41.4 cell line. **Table S4.** List of DETs upregulated in the [ABFG] subgroup vs the [CDE] subgroup of the CTC-MCC-41.5 cell lines. **Table S5.** List of DETs downregulated in the [ABFG] subgroup vs the [CDE] subgroup of the CTC-MCC-41.5 cell lines. **Table S6.** Primer sequences.

**Additional file 3: Figure S1.** Box-plots showing the expression level of a set of differentially expressed transcripts (DETs) in the CTC-MCC-41, CTC-MCC-41.4, and CTC-MCC-41.5 [ABFG] and [CDE] cell lines. Abbreviations: CTC-BT, CTC line derived before treatment initiation; CTC-AT, CTC lines derived after treatment initiation. **Figure S2.** RT-qPCR validation of the microarray data. Abbreviations: CTC-BT, CTC line derived before treatment initiation; CTC-AT, CTC lines derived after treatment initiation.

## Data Availability

The datasets used and/or analyzed during the current study are available from the corresponding author on reasonable request. The 20 publicly available Affymetrix datasets on normal colon (*n* = 5 samples), primary tumor (*n* = 5 samples), liver metastases (*n* = 5 samples) and normal liver (*n* = 5 samples) are accessible at the gene expression Omnibus (GEO) repository (https://www-ncbi-nlm-nih-gov.proxy.insermbiblio.inist.fr/geo) through the accession numbers: GSM1198032, GSM1198034, GSM1198038, GSM1198047, GSM1198050, GSM1198030, GSM1198035, GSM1198037, GSM1198046, GSM1198048, GSM1198023, GSM1198031, GSM1198033, GSM1198042, GSM1198052, GSM557089, GSM557091, GSM557093, GSM557099, GSM557107.

## Abbreviations

5-FU: 5-fluorouracile
ALDOB: Aldolase B
CDA: Cytidine deaminase
CTC: Circulating tumor cell
EMT: Epithelial to mesenchymal transition
