# Supplementary material for: Selective treatment pressure in colon cancer drives the molecular profile of resistant circulating tumor cell clones
Source: Mol Cancer. 2021 Feb 8;20:30. doi: 10.1186/s12943-021-01326-6 (PMC7869222; doi:10.1186/s12943-021-01326-6)
Supplement: Supplementary file 1 — Additional file 1. Supplementary materials and methods. [file 12943_2021_1326_MOESM1_ESM.docx]

# **Supplementary Material and Methods**

**CTC isolation and *in vitro* culture**

The nine colon CTC lines were established after CTC isolation from serial blood samples of a patient with MCC by negative enrichment using RosetteSep® (CTC Enrichment Cocktail CD36# 15167, StemCell Technologies) as previously described (10, 12).

**RNA extraction and microarray hybridization**

Total RNA from each sample was extracted with the RNeasy Mini Kit (74106-Qiagen), following the manufacturer’s instructions. The RNA quantity was determined using a NanoDrop spectrophotometer (ND-1000, Thermo Fisher Scientific), and RNA integrity using the Agilent 2100 Bioanalyzer (Agilent Technologies). Total RNA (200 ng) was used to prepare cRNA according to the Affymetrix 3’ IVT express protocol (Ref.901229). cRNA was amplified by *in vitro* transcription. Amplified RNA (aRNA) was quantified with a NanoDrop ND-1000 spectrophotometer. After fragmentation, 12 µg of labeled antisense aRNA was hybridized to HGU133plus 2.0 GeneChip arrays (Affymetrix®). In total, 13 chips (3 chips for the CTC-MCC-41 cell samples, 3 chips for the CTC-MCC-41.4 cell samples, and 1 chip for each CTC-MCC-41.5 (A-G) cell sample) were used for microarray hybridization. Microarray data were obtained and analyzed according to the minimal information about microarray experiment (MIAME) recommendations (47).

**Data processing and visualization**

After image processing with the Affymetrix GeneChip® Command Console® software, the CEL files were analyzed using the Affymetrix Expression Console™ software and normalized with the MAS5.0 algorithm by scaling each array to a target value (TGT) of 100 using the global scaling method to obtain an intensity value signal for each probe set. Gene annotation was performed with NetAffx ([http://www.netaffx.com](http://www.netaffx.com/)). A first selection of microarray data was based on the detection call. Then, transcripts with significant differential expression profiles were identified using the two-class Significance Analysis of Microarray (SAM) algorithm (48) with the Wilcoxon test and sample label permutation (n = 300). The algorithm was applied to each dataset separately and only transcripts with a significant false discovery rate (FDR) <5% and fold change (FC) ≥2 were considered. The Affymetrix Transcriptome Analysis Console (TAC) 4.0.2 and the Perseus software tools were used to construct a volcano plot, to compute the three-dimensional Principal Component Analysis (PCA), and for hierarchical clustering. The gene ontology (GO) analysis, and the top canonical pathways of differentially expressed transcripts were analyzed with the Ingenuity pathway analysis (IPA) software (QIAGEN Inc., <https://www.qiagenbioinformatics.com/products/ingenuitypathway-analysis>) (49) and the shinyGO software (50). Gene Set Enrichment Analysis (GSEA) was performed with the GSEA software (51). The interactive web-portal UALCAN that contains publicly available cancer OMICS data (17) was used to evaluate the relative expression of genes (e.g. *CDA*) in normal colon and colon adenocarcinoma samples.

**RT-qPCR validation**

The differential expression of genes identified in the microarray analysis (FC >2) was confirmed by reverse transcription-quantitative PCR (RT-qPCR). Complementary DNA (cDNA) was obtained by RT using the SuperScript® III First-Strand Synthesis Super Mix kit (18080, Invitrogen, Carlsbad, USA), according to the manufacturer’s instructions. PCR amplification was carried out using the Brillant III Ultra-Fast SYBR®Green Master Mix (600822-Agilent Technologies) on a QuantStudio 5 real-time PCR instrument (ThermoFisher, Waltham, USA). All samples were amplified in triplicate reactions and gene expression was quantified relative to the reference gene, beta-2-microglobuline (β2M), in each sample. The primer sequences are shown in **Additional file 2: Table S6**.

**Enzyme-linked immunosorbent assay (ELISA) for CDA detection**

CDA secretion was measured in culture supernatants of the nine CTC lines with the ELISA Kit for Cytidine Deaminase (CDA) (SEC366Hu, Cloud-Clone Corp.) following the manufacturer’s instructions. One million cells were cultured in 250µl of complete medium for 24h; then, supernatants were collected and centrifuged before CDA measurement by ELISA. Fresh medium was used as background control to calibrate the experiment. In parallel, the total amount of proteins was evaluated in each supernatant using the Pierce™ Rapid Gold BCA Protein Assay Kit (A53225, ThermoFisher), as recommended by the supplier, to normalize CDA results.

**Statistical Analysis**

Data analysis was performed with the GraphPad software. Differences among CTC samples were evaluated using the Student’s *t*-test, and considered significant when the *p*-value was ≤0.05. Each experiment was performed as least three times and data are shown as the mean ± SEM.
