# Supplementary material for: Selective treatment pressure in colon cancer drives the molecular profile of resistant circulating tumor cell clones
Source: Mol Cancer. 2021 Feb 8;20:30. doi: 10.1186/s12943-021-01326-6 (PMC7869222; doi:10.1186/s12943-021-01326-6)
Supplement: Supplementary file 3 — Additional file 3: Figure S1. Box-plots showing the expression level of a set of differentially expressed transcripts (DETs) in the CTC-MCC-41, CTC-MCC-41.4, and CTC-MCC-41.5 [ABFG] and [CDE] cell lines. Abbreviations: CTC-BT, CTC line derived before treatment initiation; CTC-AT, CTC lines derived after treatment initiation. Figure S2. RT-qPCR validation of the microarray data. Abbreviations: CTC-BT, CTC line derived before treatment initiation; CTC-AT, CTC lines derived after treatment initiation. [file 12943_2021_1326_MOESM3_ESM.zip › Figure S1.pptx]

## Slide 1
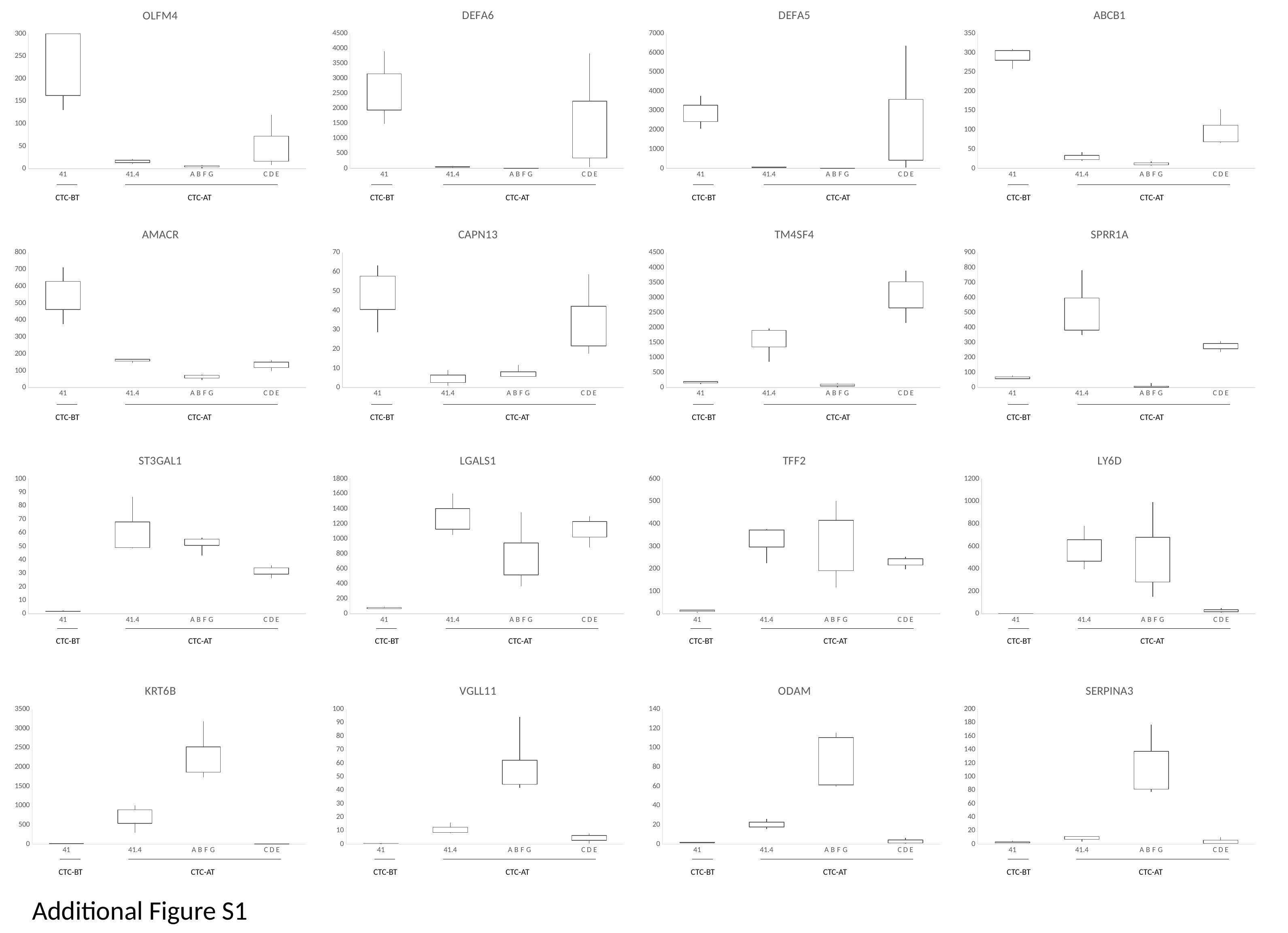

[unsupported chart]
[unsupported chart]
[unsupported chart]
[unsupported chart]
CTC-BT
CTC-AT
CTC-BT
CTC-AT
CTC-BT
CTC-AT
CTC-BT
CTC-AT
[unsupported chart]
[unsupported chart]
[unsupported chart]
[unsupported chart]
CTC-BT
CTC-AT
CTC-BT
CTC-AT
CTC-BT
CTC-AT
CTC-BT
CTC-AT
[unsupported chart]
[unsupported chart]
[unsupported chart]
[unsupported chart]
CTC-BT
CTC-AT
CTC-BT
CTC-AT
CTC-BT
CTC-AT
CTC-BT
CTC-AT
[unsupported chart]
[unsupported chart]
[unsupported chart]
[unsupported chart]
CTC-BT
CTC-AT
CTC-BT
CTC-AT
CTC-BT
CTC-AT
CTC-BT
CTC-AT
Additional Figure S1
