# Supplementary material for: Selective treatment pressure in colon cancer drives the molecular profile of resistant circulating tumor cell clones
Source: Mol Cancer. 2021 Feb 8;20:30. doi: 10.1186/s12943-021-01326-6 (PMC7869222; doi:10.1186/s12943-021-01326-6)
Supplement: Supplementary file 3 — Additional file 3: Figure S1. Box-plots showing the expression level of a set of differentially expressed transcripts (DETs) in the CTC-MCC-41, CTC-MCC-41.4, and CTC-MCC-41.5 [ABFG] and [CDE] cell lines. Abbreviations: CTC-BT, CTC line derived before treatment initiation; CTC-AT, CTC lines derived after treatment initiation. Figure S2. RT-qPCR validation of the microarray data. Abbreviations: CTC-BT, CTC line derived before treatment initiation; CTC-AT, CTC lines derived after treatment initiation. [file 12943_2021_1326_MOESM3_ESM.zip › Figure S2.pptx]

## Slide 1
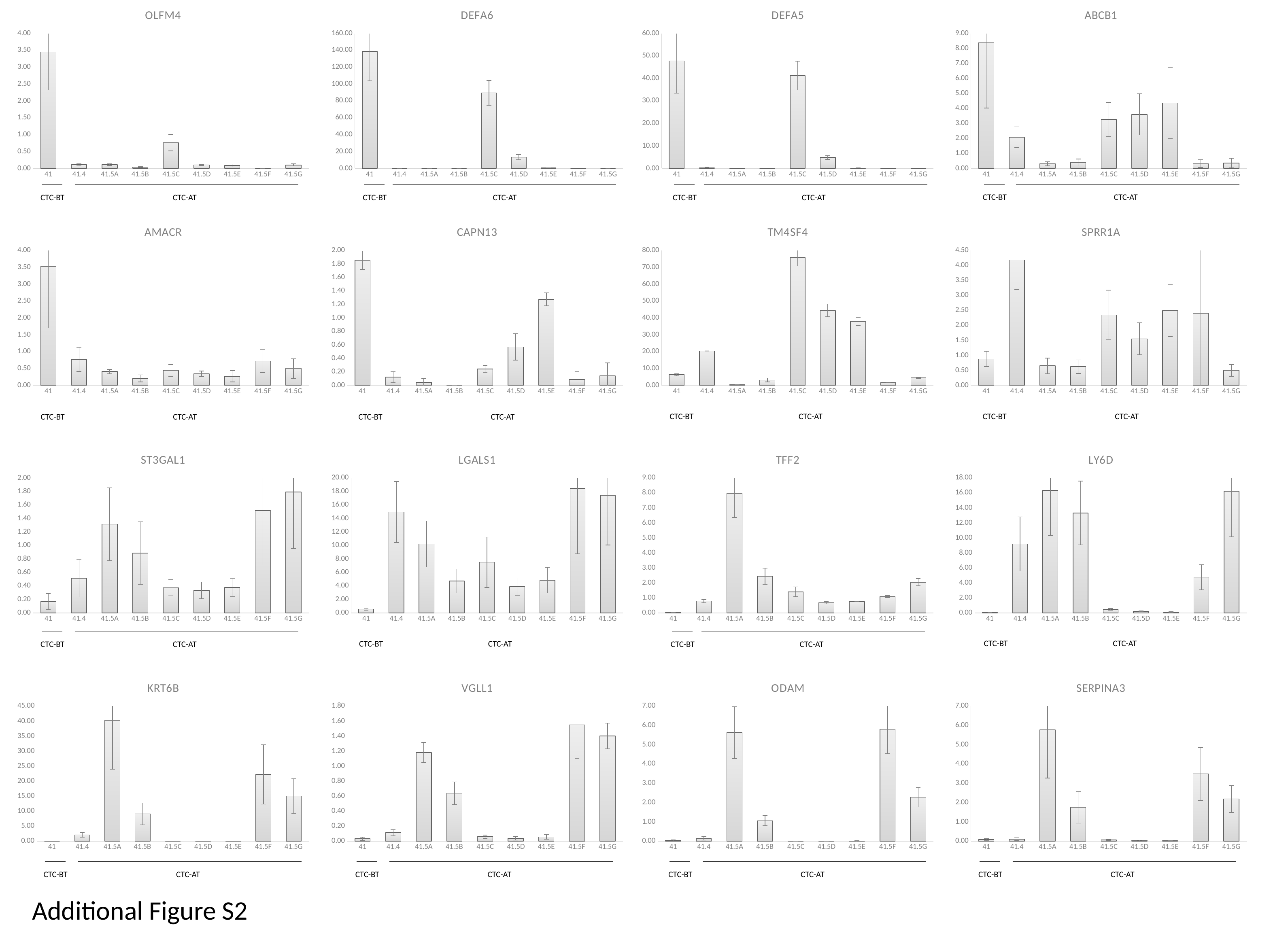

### Chart:
| Category | OLFM4 |
|---|---|
| 41 | 3.4525215610331514 |
| 41.4 | 0.10844101582138853 |
| 41.5A | 0.11161494665848397 |
| 41.5B | 0.035348834870686796 |
| 41.5C | 0.76438156955684 |
| 41.5D | 0.10265968437992871 |
| 41.5E | 0.08379659144235925 |
| 41.5F | 5.812119916760502e-07 |
| 41.5G | 0.09910950781506961 |
### Chart:
| Category | DEFA6 |
|---|---|
| 41 | 138.76649492572605 |
| 41.4 | 0.14948059742715966 |
| 41.5A | 0.03688234148966172 |
| 41.5B | 0.06083365347276537 |
| 41.5C | 89.52088894334914 |
| 41.5D | 13.357107229919855 |
| 41.5E | 0.47369278126648195 |
| 41.5F | 5.812119916760502e-07 |
| 41.5G | 0.05818643903950468 |
### Chart:
| Category | DEFA5 |
|---|---|
| 41 | 47.776730734307286 |
| 41.4 | 0.24722487990560324 |
| 41.5A | 5.364482495766013e-07 |
| 41.5B | 5.64759061295973e-07 |
| 41.5C | 41.19313101719258 |
| 41.5D | 4.848718523849412 |
| 41.5E | 0.1335392615428685 |
| 41.5F | 5.530897436017638e-07 |
| 41.5G | 6.195361862493996e-07 |
### Chart:
| Category | ABCB1 |
|---|---|
| 41 | 8.384221829221767 |
| 41.4 | 2.073392937823055 |
| 41.5A | 0.3174876567777702 |
| 41.5B | 0.3941781711523082 |
| 41.5C | 3.2659123967018764 |
| 41.5D | 3.594241316564807 |
| 41.5E | 4.363500072077341 |
| 41.5F | 0.31242290980968573 |
| 41.5G | 0.36026941441117416 |CTC-BT
CTC-AT
CTC-BT
CTC-AT
CTC-BT
CTC-AT
CTC-BT
CTC-AT
### Chart:
| Category | AMACR |
|---|---|
| 41 | 3.5322071290413355 |
| 41.4 | 0.7703377026129715 |
| 41.5A | 0.4138701254453434 |
| 41.5B | 0.21119648907025365 |
| 41.5C | 0.4463189312251059 |
| 41.5D | 0.3432153758432542 |
| 41.5E | 0.26904943563565975 |
| 41.5F | 0.7221774326854055 |
| 41.5G | 0.5029046228886264 |
### Chart:
| Category | CAPN13 |
|---|---|
| 41 | 1.8536248442529555 |
| 41.4 | 0.12306011772977203 |
| 41.5A | 0.044807814902058735 |
| 41.5B | 5.16285357558613e-07 |
| 41.5C | 0.24527021586790856 |
| 41.5D | 0.5703427137951452 |
| 41.5E | 1.276435215105724 |
| 41.5F | 0.08692697118396954 |
| 41.5G | 0.14250598624273605 |
### Chart:
| Category | TM4SF4 |
|---|---|
| 41 | 6.410807353789248 |
| 41.4 | 20.401335183523017 |
| 41.5A | 0.3594868590491768 |
| 41.5B | 3.189721941147584 |
| 41.5C | 75.79523377505892 |
| 41.5D | 44.43665268895963 |
| 41.5E | 37.96171999346118 |
| 41.5F | 1.680934363639351 |
| 41.5G | 4.4200798918700075 |
### Chart:
| Category | SPRR1A |
|---|---|
| 41 | 0.8812291665939594 |
| 41.4 | 4.184565804593134 |
| 41.5A | 0.6548815111313764 |
| 41.5B | 0.6274173144307859 |
| 41.5C | 2.346073743845704 |
| 41.5D | 1.5558041591573228 |
| 41.5E | 2.493368009951937 |
| 41.5F | 2.412409141723358 |
| 41.5G | 0.49949141338495306 |CTC-BT
CTC-AT
CTC-BT
CTC-AT
CTC-BT
CTC-AT
CTC-BT
CTC-AT
### Chart:
| Category | ST3GAL1 |
|---|---|
| 41 | 0.1681064521876269 |
| 41.4 | 0.5132848815117098 |
| 41.5A | 1.3150248934261286 |
| 41.5B | 0.8876085144611078 |
| 41.5C | 0.3746820704921712 |
| 41.5D | 0.33336690376091754 |
| 41.5E | 0.3776808717098064 |
| 41.5F | 1.5150864304321832 |
| 41.5G | 1.7904679437199686 |
### Chart:
| Category | LGALS1 |
|---|---|
| 41 | 0.5541159593560124 |
| 41.4 | 14.948162376249286 |
| 41.5A | 10.219794008236212 |
| 41.5B | 4.717626286606424 |
| 41.5C | 7.500977518752997 |
| 41.5D | 3.878976950412998 |
| 41.5E | 4.8573227835738875 |
| 41.5F | 18.453873144017262 |
| 41.5G | 17.418686900829314 |
### Chart:
| Category | TFF2 |
|---|---|
| 41 | 0.027910934568190743 |
| 41.4 | 0.7862462484257456 |
| 41.5A | 7.964356464554419 |
| 41.5B | 2.4429569860179208 |
| 41.5C | 1.399186130568052 |
| 41.5D | 0.6746513959576385 |
| 41.5E | 0.7505114115020076 |
| 41.5F | 1.0877332803928217 |
| 41.5G | 2.047507582063008 |
### Chart:
| Category | LY6D |
|---|---|
| 41 | 0.06405224734098919 |
| 41.4 | 9.18774920453629 |
| 41.5A | 16.324917809373215 |
| 41.5B | 13.331891076462066 |
| 41.5C | 0.46278832628802774 |
| 41.5D | 0.21106121135417152 |
| 41.5E | 0.0977604867602324 |
| 41.5F | 4.772070476823218 |
| 41.5G | 16.212271966086163 |CTC-BT
CTC-AT
CTC-BT
CTC-AT
CTC-BT
CTC-AT
CTC-BT
CTC-AT
### Chart:
| Category | KRT6B |
|---|---|
| 41 | 2.645289515617937e-07 |
| 41.4 | 2.103402778655244 |
| 41.5A | 40.252606543834574 |
| 41.5B | 9.080567837198329 |
| 41.5C | 3.0319395238337605e-07 |
| 41.5D | 2.3703133348150942e-07 |
| 41.5E | 2.670365060995549e-07 |
| 41.5F | 22.234176828454604 |
| 41.5G | 15.03796701607255 |
### Chart:
| Category | VGLL1 |
|---|---|
| 41 | 0.03169496401498669 |
| 41.4 | 0.11255469675786556 |
| 41.5A | 1.1818388372518729 |
| 41.5B | 0.6389012978462132 |
| 41.5C | 0.058425861308806414 |
| 41.5D | 0.0380150829390151 |
| 41.5E | 0.056050853618877274 |
| 41.5F | 1.5519316846532185 |
| 41.5G | 1.4029636650118562 |
### Chart:
| Category | ODAM |
|---|---|
| 41 | 0.04417887105767083 |
| 41.4 | 0.13923216600613986 |
| 41.5A | 5.624792224112905 |
| 41.5B | 1.0628245045665585 |
| 41.5C | 3.0319395238337605e-07 |
| 41.5D | 2.3703133348150942e-07 |
| 41.5E | 0.006739617553329714 |
| 41.5F | 5.800581122353454 |
| 41.5G | 2.270926225549182 |
### Chart:
| Category | SERPINA3 |
|---|---|
| 41 | 0.07739798082647796 |
| 41.4 | 0.1089070250676955 |
| 41.5A | 5.763907513620698 |
| 41.5B | 1.7510818206185463 |
| 41.5C | 0.061198751747317835 |
| 41.5D | 0.029120462901311994 |
| 41.5E | 0.016091540518604248 |
| 41.5F | 3.4918690987378067 |
| 41.5G | 2.189048590657014 |CTC-BT
CTC-AT
CTC-BT
CTC-AT
CTC-BT
CTC-AT
CTC-BT
CTC-AT
Additional Figure S2
